# Identification and assessment of health-related quality of life issues in patients with sporadic desmoid-type fibromatosis: a literature review and focus group study

**DOI:** 10.1007/s11136-018-1931-3

**Published:** 2018-07-16

**Authors:** Milea J. M. Timbergen, Lonneke V. van de Poll-Franse, Dirk J. Grünhagen, Winette T. van der Graaf, Stefan Sleijfer, Cornelis Verhoef, Olga Husson

**Affiliations:** 1 Department of Surgical Oncology, Erasmus MC Cancer Institute Rotterdam, ‘s-Gravendijkwal 230, Room BE-428, 3015 CE Rotterdam, The Netherlands; 2 Department of Medical Oncology, Erasmus MC Cancer Institute Rotterdam, ‘s-Gravendijkwal 230, Room BE-428, 3015 CE Rotterdam, The Netherlands; 3 Department of Research, Netherlands Comprehensive Cancer Organisation (IKNL), Eindhoven, The Netherlands; 4 Division of Psychosocial Research and Epidemiology, The Netherlands Cancer Institute, Amsterdam, The Netherlands; 5 Department of Medical and Clinical Psychology, Tilburg University, Tilburg, The Netherlands; 6 Department of Medical Oncology, Radboud University Medical Center, Nijmegen, The Netherlands; 7 Division of Clinical Studies, Institute of Cancer Research and Royal Marsden NHS Foundation Trust, London, UK

**Keywords:** Desmoid-type fibromatosis, Health-related quality of life, Focus group, Literature review

## Abstract

**Purpose:**

Sporadic desmoid-type fibromatosis (DTF) is a rare, chronic, non-metastasising, disease of the soft tissues. It is characterised by local invasive and unpredictable growth behaviour and a high propensity of local recurrence after surgery thereby often having a great impact on health-related quality of life (HRQL). This study aims to review currently used HRQL measures and to asses HRQL issues among DTF patients.

**Methods:**

A mixed methods methodology was used consisting of (1) a systematic literature review, according to the PRISMA guidelines (2009), using search terms related to sporadic DTF and HRQL in commonly used databases (e.g. Embase, Medline Ovid, Web of science, Cochrane Central, Psyc Info, and Google scholar), to provide an overview of measures previously used to evaluate HRQL among DTF patients; (2) focus groups to gain insight into HRQL issues experienced by DTF patients.

**Results:**

The search strategy identified thirteen articles reporting HRQL measures using a wide variety of cancer-specific HRQL tools, functional scores, symptom scales (e.g. NRS), and single-item outcomes (e.g. pain and functional impairment). No DTF-specific HRQL tool was found. Qualitative analysis of three focus groups (6 males, 9 females) showed that participants emphasised the negative impact of DTF and/or its treatment on several HRQL domains. Six themes were identified: (1) diagnosis, (2) treatment, (3) follow-up and recurrence, (4) physical domain, (5) psychological and emotional domain, and (6) social domain.

**Conclusion:**

A DTF-specific HRQL tool and consensus regarding the preferred measurement tool among DTF patients is lacking. Our study indicates that HRQL of DTF patients was negatively affected in several domains. A DTF-specific HRQL measure could improve our understanding of short- and long-term effects and, ideally, can be used in both clinic and for research purposes.

## Introduction

Desmoid-type fibromatosis (DTF) is a soft tissue tumour that arises from musculoaponeurotic structures. It is incapable of metastasising and is often described as a benign tumour in clinical practice. However, due to its local aggressive behaviour and its known tendency of local recurrence after initial surgical resection, it is categorised as a borderline tumour [1]. Desmoid-type fibromatosis is rare, with a reported incidence of 5.4 new cases per million persons per year in the Dutch population [2]. Symptoms vary, depending on tumour location and size, and can be very severe. Roughly two types can be distinguished: sporadic DTF with extra-abdominal or abdominal wall tumour formation and familial adenomatous polyposis (FAP)-related DTF with intra-abdominal tumour formation [3, 4].

The aetiology of sporadic DTF remains doubtful although a history of trauma has been reported, as well as specific hormonal status (such as pregnancy) and genetic predisposition [5–8]. With local recurrence rates up to 50%, potential treatment benefits and adverse effects of treatment should be considered carefully [9–11]. Nowadays, active surveillance is recommended in asymptomatic patients, while treatment options for symptomatic patients include surgical resection, radiation therapy, and systemic therapy [12–16]. Determination of treatment effectiveness is currently mainly evaluated by tumour size or recurrence free survival [11, 17, 18]. Although such end-points can be appropriate in malignant diseases, the unpredictable growth behaviour including spontaneous regression and the low mortality rate of sporadic DTF renders such outcomes less appropriate for this borderline disease [16]. Consequently, the question rises whether health-related quality of life (HRQL) assessment could be a more appropriate outcome measure in DTF [10, 14, 19, 20]. The definition of HRQL is “a patients’ evaluation of the impact of a health condition and its treatment on all relevant aspects of life”. Patient-reported outcome measures (PROMs) can be used to measure HRQL with various purposes: screening tools, method for identifying patient preferences, to guide clinicians for informed decision making, to improve patient-provider communication, and to assess the efficacy of treatments in the context of clinical trials [21]. In DTF, few researchers have sought to understand patient’s perceptions on the disease, and HRQL is not (yet) widely accepted as an appropriate outcome measure. The aim of this mixed-method study is to explore currently used HRQL tools and identify HRQL issues of DTF patients.

## Methods

### Literature review

The literature review was conducted in accordance with the PRISMA guidelines [22]. A systematic literature search with terms related to sporadic DTF and HRQL (Appendix 1) was conducted by an expert research librarian on 6 November 2017 to identify HRQL tools currently used among DTF patients. No language or publication limitations were applied. Used databases were Embase, Medline Ovid, Web of science, Cochrane Central, Psyc Info, and Google scholar. The resulting publications were analysed using inclusion and exclusion criteria at two levels: title/ abstract (1) and full text (2) by two reviewers (MJMT and OH). Data from papers that met the inclusion criteria at full-text level were extracted for final inclusion by one reviewer [MJMT] (Appendix 2). Corresponding authors were contacted in case of lack of availability of full text, and three authors granted our request. Variables that were identified in included papers were number of patients, number of patients for which PROMs were available, tumour location, treatment, PROM outcome pre-treatment, and PROM outcome post-treatment. The outcome of each study was reported according to the specific PROM used in the study.

### Patient recruitment

To identify the HRQL issues of DTF patients, focus group sessions were organised. Patients diagnosed with sporadic DTF were recruited from the Erasmus Medical Centre (MC) in Rotterdam, the Netherlands. As FAP-associated DTF patients are also confronted with many other issues compared to patients with sporadic DTF, these patients were excluded. Eligible patients were diagnosed with DTF, regardless of their stage of disease (e.g. pre-treatment or during follow-up), previous or current treatments, and site of disease. Additionally, they had to be above the age of 18 at the time of the focus group and participation required sufficient Dutch language skills. Patients with a recent diagnosis of cancer were excluded since this diagnosis might influence their HRQL. Potential participants were approached by telephone, with a maximum of four attempts to explain the study objectives and received a written invitation and information letter. In total, three focus groups were organised in July and August 2017: one with male participants, one with female participants, and one mixed sex group. The decision to organise separate sessions for both sexes was based on the assumption that patients would be more likely to share personal experiences or feelings with the same sex. The third, mixed sex group was organised separately because of logistic reasons. The focus group sessions took place in the Erasmus MC. Written informed consent (including permission for making field notes and audio recording for anonymous processing) and background information was obtained at the start.

### Data collection

**Fig. 1:**
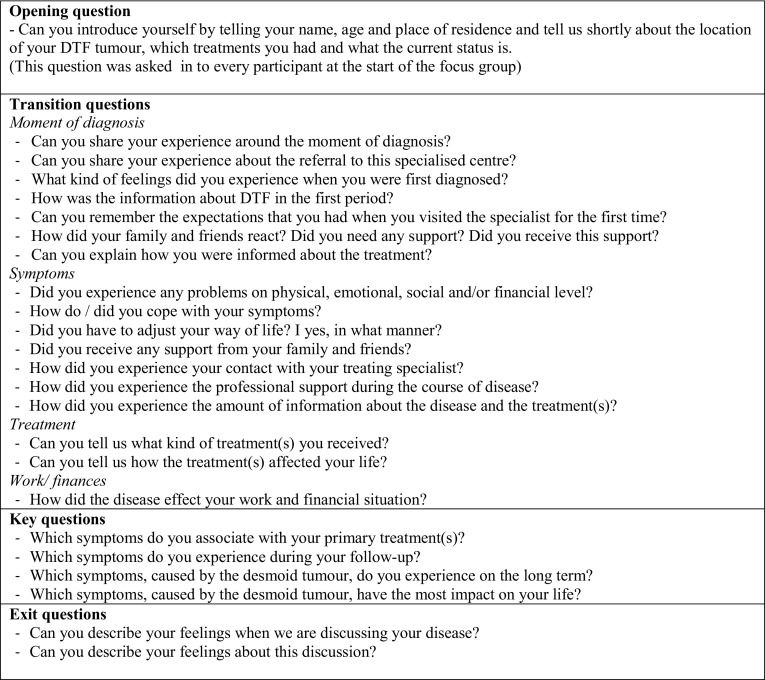
Focus group guideline

The focus group sessions were supervised by the first author [MJMT]; a second independent researcher kept written records and was not actively involved in the discussions. A pre-prepared protocol, based on the protocol of Husson et al. (2018, manuscript submitted) was used for guidance (Fig. 1).

Participants received a brief introduction with the explanation of the study objectives and an opening question for introduction was answered by each participant. Next, pre-prepared exploratory questions developed specific for the objectives of this study were asked to encourage conversation and discussion. Transition questions were asked to explore several aspects of HRQL issues around the time of diagnosis, treatment, and follow-up. The focus groups lasted 1.5–2 h, and an exit question was used to terminate the focus group. Participants received an evaluation form and 15 euro gift certificate in order to express our appreciation for their participation. Focus group sessions were audio recorded, and transcribed by the first author [MJMT]. ATLAS.ti 8.0^©^ (Scientific Software Development GmbH, Berlin, Germany) was used for generating codes for themes and subthemes. The field notes were used as complementary data to transcripts as they described non-verbal communication of participants. Data were ordered into relevant code terms and then categorised into themes by two researchers [MJMT and OH] and analysed independently. Consensus was reached through continuous discussion. Relevant quotes of focus group participants were selected to support findings.

Approval from the Medical Ethics Committee of Erasmus MC in Rotterdam, the Netherlands was obtained for this study (file number MEC-2017-269). All patients gave written informed consent before the start of the focus groups and patient’s anonymity and confidentiality were ensured throughout the study by the use of study codes replacing identifying information. Only the first author had direct access to the digital record of study codes and patient information.

## Results

### Literature review

A systematic literature search (Appendix 1) showed 3114 articles after deduplication. In total, 3067 articles were excluded based on title or abstract. Full-text reviewing took place for the remaining 47 articles excluding another 34 articles (flow chart Appendix 2). Thirteen articles describing seven validated scoring systems were identified. No DTF-specific questionnaires were identified in this literature review (Table 1).

**Table 1:** Review of literature Patient-Reported Outcome Measurements (PROMs)

Score 1	Score 2/additional interests	Ref.	N (N-PROM)	Location	T	Outcome pre-treatment	Outcome post-treatment
DASH	**–**	[26]	14 (1)^a^	UE	SG	NA	*n* = 1 DASH 38 (after interthoracoscapular amputation)^a^
	MSTS score	[24]	12 (1)^a^	UE	SG	NA	*n* = 1^a^ MSTS 50%^b^ (pain 2, function 1, acceptance 2, hand positioning 4, dexterity 3, lifting 3), DASH 62.5^b^
Enneking score/MSTS score	**–**	[27]	21	UE	SG BT	NA	Mean MSTS 79%^b^ (range 57–97%) *n* = 10 (48%) excellent; *n* = 10 (48%) good; *n* = 1 (4%) medium
	Pain functional impairment	[30]	7	UE	SG ARTx CT	NA	Mean MSTS 73% (range 36–90%), *n* = 5 moderate functional impairment of the shoulder, *n* = 1 deficit flexion and extension of the elbow. *n* = 2 pain with use of medication
	TESS	[28]	14 (1)^a^	UE	SG	NA	*n* = 1 MSTS 50 (pain 3, function 2, acceptance 5, hand positioning 0, dexterity 5, lifting 0), TESS 62^a^
EORTC QLQ-C30		[19]	14	AW	SG	NA	Mean global health status^b^: 97 (± 5.9); physical functioning 93 (± 11.1); role functioning 89 (± 16.7); emotional functioning 87 (± 19.1); cognitive functioning 94 (± 8.3); social functioning 93 (± 14.7)
MDASI	**–**	[32]	17	AS	GSI	NA	Mean symptom severity: partial responders (*n* = 5): 1.65 point improvement, stable disease (*n* = 5): 0.8 point improvement, no response/dropout (*n* = 7) 0.04 point improvement
Modified Johnstone scale	**–**	[34]	40 (24)	AS	SG ARTx CT	*n* = 24 amputation required (grade 0): *n* = 1 (4%), severe functional deficit (grade 1): *n* = 1 (4%), major functional limitations (grade 2): *n* = 4 (17%), mild functional limitations (grade 3): *n* = 7 (29%), no functional limitations (grade 4): *n* = 9 (38%), NA: *n* = 2 (8%)	*n* = 24 grade 0: *n* = 5 (21%); grade 1: *n* = 0; grade 2: *n* = 6 (25%); grade 3: *n* = 3 (13%); grade 4: *n* = 8 (33%) not recorded: *n* = 2 (8%)
NRS	**–**	[37]	15 (6)	EA	MRgFUS CT CA	*n* = 6 NRS 7.5 (± 1.9) (worst daily NRS); *n* = 6 NRS 6 (± 2.3) (average daily NRS)	*n* = 6 NRS 2.7 (± 2.6) (worst daily NRS); *n* = 6 NRS 1.3 (± 2) (average daily NRS)
	**–**	[38]	44	AS	ST	Median NRS 6 (IQR 2–7) group B: median NRS 7/10; *n* = 7 moderate pain, *n* = 17 severe pain; group C: *n* = 8 severe pain^c^	Group B *n* = 12 (75%) pain improvement, *n* = 1 (6%) pain worsening, *n* = 3 (19%) stable symptoms; group C: *n* = 8 (100%) pain relief^c^
Other scores	Functional outcome	[43]	21	HN	SG ARTx	Asymptomatic *n* = 14 (62%), neurologic symptoms *n* = 8 (38%)	*n* = 8 (38%) good, *n* = 13 (62%) persistent functional problems (motor (*n* = 7), paraesthesia (*n* = 4))
	Functional impairment	[41]	106	AS	SG ARTx RTx ST	NA	0–1 T: 23% functional impairment: moderate *n* = 13, major *n* = 2; 2 T: 56% functional impairment: moderate *n* = 8, major *n* = 7; ≥ 3 T: 74% functional impairment: moderate *n* = 7, major *n* = 10
	Pain, functional, impairment cosmetic outcome	[42]	12 (7)	EA	CT	*n* = 7 pain; *n* = 7 functional limitation; *n* = 3 cosmesis	*n* = 6 pain relief; *n* = 3 partial improvement of function, *n* = 4 restore of normal function; *n* = 2 improvement of cosmetic outcome

*PROM* Patient-Reported Outcome Measurement, *Ref*. Reference, *N* Number of patients, *N-PROM* Number of patients with DTF and available patient-reported outcomes, *T* Treatment, *DASH* Disabilities of the Arm, Shoulder, and Hand, *Enneking*/*MSTS* *score* Enneking score adopted by the Musculoskeletal Tumour Society, *EORTC QLQ-C30* The European Organisation for Research and Treatment of Cancer quality of life questionnaire C30, *TESS* Toronto Extremity Salvage score, *MDASI* MD Anderson symptom Inventory, *NRS* Numerical Rating Scale, *PD* Progressive disease, *NA* Not applicable, *AS* all sites, *EA* extra-abdominal, *AW* abdominal wall, *IA* intra-abdominal, *LE* lower extremities, *HN* head and neck, *UE* upper extremities, *ACT* adjuvant chemotherapy, *ARTx* adjuvant radiotherapy, *BT* brachytherapy, *CA* cryoablation, *CT* chemotherapy, *GSI* γ-secretase inhibitor, *MRgFUS* magnetic resonance-guided focused ultrasound, *NSG* no-surgery, *SG* surgery, *RTx* radiotherapy

^a^Desmoid-type fibromatosis and soft tissue sarcoma combined, reported outcome for subgroup of DTF

^b^At most recent reported follow-up

^c^ *Group A* radiological progressive disease (PD), *Group B* symptomatic deterioration, and *Group C* radiologically PD and symptomatic deterioration

The Disabilities of the Arm, Shoulder and Hand (DASH) score is a 30-item questionnaire designed to evaluate disability of the upper limb region by measuring symptoms and physical functions with 5 response options and higher scores reflecting greater disability [23–26]. The Enneking/Musculoskeletal Tumor Society (MSTS) score comprises six categories: pain, function, and emotional acceptance of both lower and upper extremities, support, walking, and gait of the lower extremities, and hand positioning, dexterity, and lifting ability in the upper extremity, for which patients have to assign values ranging from 0 to 5 points. Higher values indicate better functioning [24, 27–30]. The European Organisation for Research and Treatment of Cancer quality of life questionnaire C30 (EORTC QLQ-C30) is a 30-item, cancer-specific questionnaire designed for evaluating quality of life incorporating five functional scales, symptom scales, and global health and quality of life scales [19, 31]. The MD Anderson Symptom Inventory (MDASI) measures the severity of 13 cancer-related symptoms experienced by the patient during the previous 24 h. The score rates symptoms on an 11-point scale; higher scores reflect more severe symptoms [32, 33]. The (modified) Johnstone scale provides a functional grading system with grades ranging from 0 to 4; higher scores reflect fewer limitations [34, 35]. The Numerical Rating Scale (NRS) is used for self-reporting subjective conditions, currently in use for several symptoms. Symptoms are rated on a 0–10 scale; higher scores reflect more severe symptoms [36–38]. The Toronto Extremity Salvage score (TESS) is internationally used for measuring functional outcome and physical disability in patients with extremity tumours undergoing limb preservation surgery. This questionnaire consists of 29 (upper extremity) or 30 (lower extremity) questions regarding daily activities. Each item is rated on a scale from 1 to 5; higher values represent better function [28, 39, 40].

Other identified measures and questionnaires included items related to functional impairment, pain, and cosmetic outcome (Table 1) [41–43].

### Focus group

In total, 45 patients were approached to participate; 22 patients agreed to receive written information, and 15 patients could not be reached by telephone. Reasons for refusal included not willing to participate in a group experience but willing to do a personal interview, not available at pre-set dates, language barrier, or not willing to participate because of minimal symptoms. A total of 15 patients participated in the focus groups. The first group consisted of five female participants with a median age of 37 years (range 25–60 years), the second group consisted of five male participants with a median age of 62 (range 37–75 years), and the third group was a mixed sex group with a median age of 37 years (range 36–53 years). Participants differed in age at diagnosis, education level, and treatment (Table 2). None of the participants knew another person with the same condition before the focus group. Most participants were treated surgically (*n* = 8) or received a conservative management (*n* = 4). Three participants received a combination of therapies. A minority of the participants sought support in the paramedic field (e.g. physiotherapist, occupational therapist, social worker, and dietician).

**Table 2:** Characteristics of fifteen focus group participants

	Number of patients (%)	Age in years (range)
Sex		
Male	9 (60%)	
Female	6 (40%)	
Age at time of focus group		
Median (range) years		46 (25–75)
Age at time of diagnosis		
Median (range) years		43 (16–75)
Marital status		
Single	3 (20%)	
Married	9 (60%)	
Partnership	2 (13%)	
Windowed	0 (0%)	
Divorced	1 (7%)	
Nationality		
Dutch	14 (93%)	
Other	1 (7%)	
Highest completed education		
Elementary education	1 (7%)	
Secondary education	2 (13%)	
Middle-level applied education	3 (20%)	
Higher professional education	6 (40%)	
Scientific education (university)	1 (7%)	
Missing value	2 (13%)	
Current paid employment		
Yes	8 (53%)	
No	5 (33%)	
Retired	2 (13%)	
Familiar with DTF before diagnosis		
Yes	0 (0%)	
No	15 (100%)	
Location of DTF		
Head/neck	1 (7%)	
Upper extremity/shoulder	2 (13%)	
Thoracic wall	0 (0%)	
Abdominal wall	4 (27%)	
Back	1 (7%)	
Retroperitoneal/intra-abdominal	2 (13%)	
Hip/pelvis/gluteal region	2 (13%)	
Lower extremity	3 (20%)	
Received treatment(s)		
Conservative management	4 (27%)	
Surgery	8 (53%)	
Radiation therapy	0 (0%)	
Systemic therapy	0 (0%)	
Combination of therapies^a^	3 (20%)	
Contact with healthcare professionals		
Physiotherapist/occupational therapist	5	
Dietician	1	
Social worker	2	
Psychologist	1	
Pain specialist	1	
Home care/nursing care	1	
Other^b^	1	
Self-reported symptoms^c^		
Lump with obvious growth	10	
Pain	3	
Tumour complains during daily activities	8	
Functional limitations (before treatment)	3	
Self-reported medical history^c^		
Surgery related desmoid	6	
Desmoid related to hormonal status	3	

^a^ *n* = 1 surgical resection with post-operative radiotherapy, *n* = 1 surgical resection, radiotherapy, and isolated limb perfusion (ILP), *n* = 1 surgical resection (with final amputation of the lower leg, radiotherapy, ILP, hormonal therapy, experimental chemotherapy)

^b^Lymphatic therapy

^c^Obtained during the focus group sessions as reported by the patients

### Qualitative analysis

HRQL issues were categorised into six themes: (1) diagnosis, (2) treatment, (3) follow-up and recurrence, (4) physical domain, (5) psychological and emotional domain, and (6) social domain. The themes were further categorised into subthemes. An overview of themes, subthemes, key issues, and quotes is provided in Table 3.

**Table 3:** Themes, subthemes, key issues and quotes of three focus group sessions

Themes	Subthemes	Key issues	Quotes
Diagnosis	Uncertainties about diagnosis	Broad differential diagnosis, lack of knowledge about DTF creating feelings of uncertainty and anxiety	“sent from one specialist to another”
	Diagnosis	Referral to specialized centre is considered to be time consuming	“the feeling of insecurity, the fear of dying”
	Information about DTF	Borderline entity, presented as a “benign tumour” with an aggressive clinical course in some cases	“‘it’s a tumour and that is a disastrous scenario”
	Need for information about DTF	Lack of knowledge about DTF of treating physicians in regional hospitals	“I took a whole different scenario into account”
		Lack of up to date information for DTF patienss and their relatives	“you have cancer”
			“we can’t help you”
			“I think we have to amputate your arm”
			“to me it is frustrating, this is a benign disease, but the more you read, the more information you receive, the more you find out about its aggressiveness and invasiveness, so for me this is a malignancy”
Treatment	Treatment	Lack of uniformity in treatment between hospitals	“I am glad that the surgeon took it out”
		Shared decision making, patient autonomy	“the surgeon said: I don’t want to operate because if I do, I’m not sure what I’m going to find”
Follow-up & recurrence	Follow-up (concerns about) recurrence/concerns about the future	Lack of clear information about recurrences rates specific for personal situation	“you know it is possible that you might need surgery another time, but if it happens, it happens”
		Concerns about recurrence or concerns about future problems due to DTF	“I would love to have assurance that I am done with it”
			“you have a diagnosis, no prognosis”
Physical domain	Symptoms (pre-treatment/post-treatment)	Awareness for functional problems and anticipate by offering physical therapy	“the size of a tennis ball”
	Localization		“taking off my t-shirt is not easy, absolutely not”
	Medical history/co-morbidity		“I am asymmetrical after the surgery”
	Support physical therapy		“It took 4–6 months to be ready to practice with a prosthesis, but this leg was pretty messed up because of all the treatments”
	Self-image/cosmetic		
Psychological/emotional domain	Coping strategy Lifestyle changes Emotional& psychological consequences Psychological support	Awareness for psychological or emotional issues and anticipate by offering psychologic therapy	“this is part of my pathway in life”
			“you learn to deal with this functional limitation; you just have to changes things”
			“if this is the worst scenario, I am okay with it”
			“as long as you don’t know, you can worry about it, but it will do no good”
Social domain	Education/financial/employment	Interest for impact on situation on family members of DTF patients	“my family had more difficulty with the surgery than I did”
	Social support/support of family		“everyone is relieved because it’s benign; yes that’s what I thought the first time. Sometimes I find that difficult, because that is easy to say for people not living with a tumour in their abdomen”
			“the social pictures has changed, people I went to college with are more advanced in life, I’m standing still in life”
			“I had to move to a ground floor apartment”

### Diagnosis

Almost all participants reported feelings of uncertainty and anxiety of having cancer during the period of waiting on their final diagnosis. They described this as having a great impact on their overall life. Upon diagnosis, feelings of relief are described due to the borderline nature of this disease. Participants with more symptoms and a more aggressive clinical course of DTF mentioned being frustrated about underestimation of the consequences since the disease is categorised as a borderline tumour and can act in a more malignant way with sometimes severe sequelae compared to benign tumours. The opinion on receiving information about DTF varied among participants. Some participants felt they did not receive enough information from their treating physician, some participants searched for more information on internet or asked their general practitioner, and some deliberately did not search on the internet because of fear to find unpleasant information. Most participants agreed that the amount and depth of information they found in general was not satisfying. This observation was substantiated by multiple questions from participants about DTF during the group sessions.

### Treatment

Participants with minor symptoms and solely treated with surgery reported being glad or relieved that the tumour was removed as they had the feeling that it “did not belong to their body”. One participant with major symptoms from an intra-abdominal tumour felt that surgery was the only treatment option, but feared for a stoma or dying during surgery. Participants with a conservative management reported to be satisfied since they had minor symptoms and potentially mutilating surgery could be avoided.

### Follow-up and recurrence

A common theme in the qualitative study was fear of recurrence or worries about the future and future health. Not all participants were correctly informed about the risk on local recurrence. Feelings of uncertainty remained present during follow-up because of the knowledge that the tumour may be able to recur. One participant with DTF localised in the lower extremity reported struggle with weakness in the leg due to previous treatments, which made her fearful of the future.

### Physical domain

The most common symptoms before diagnosis are described in Table 2. Complications of treatment included infection of the surgical wound and severe neuropathic pain due to nerve damage. Residual issues after treatment regarded scars, being asymmetrical, having function restrictions, oedema, stiffness, lack of sensibility, and muscle weakness. One participant used a wheelchair and crutches due to a lower leg amputation, and another patient used an electric wheelchair due to severe neuropathic pain after being treated surgically. One participant reported that physical therapy was not offered to her, but in retrospect she would have appreciated it since she experiences weakness of the affected limb.

### Emotional/psychological domain

Participants expressed that “they felt they did not have a choice” and “they will face the situation as it comes” and learned how to deal with their problems over time. Life-style changes included minor adjustments because of functional limitations and major adjustments including movement to a ground floor apartment. One participant reported that DTF restricted her from having another child, which had a major impact on her family. One participant reported a low self-esteem and problems with body image due to scars. Another participant reported the feeling that he missed out on starting a family because of extensive treatments which started at a young age. One participant was treated by a psychologist. Several participants stressed that they felt differently about life after diagnosis and stated to be more grateful for their life compared to the time before the diagnosis.

### Social domain

Participants reported that DTF had influenced their working life, as they had to stop working temporarily after treatment. This period ranges from a couple of weeks to two years and in one case not being able to work at all. Participants reported that the uncertainty during the time of diagnosis and the fear of cancer influenced their family life. Several participants mentioned to downstage their problems since they did not want to be a burden to their families or they wanted to protect their loved ones. One participant reported that social relationships changed after the diagnosis. Some friendships became closer and some friendships had ended due to lack of support. She specifically mentioned that her friends paid less attention to her disease and health status because of the term ‘benign disease’ which implies minor disease-related issues or short course of disease.

## Discussion

With this study, we aimed to gain more insight in HRQL issues and currently used HRQL tools in the setting of DTF. The results of this study can be seen as the first step towards developing a disease-specific HRQL tool that can be used in clinical practice or research. The literature review identified several non-disease-specific HRQL tools; no tool currently exists that assesses all issues relevant for DTF patients. Functional scores like the DASH score [26], the Enneking score/MSTS [24, 27, 28, 30], the TESS [28], and the Johnstone scale [34] are used for extremity diseases but are not suitable for patients who have sites of disease other than the extremities. Symptoms scores including the MDASI score [32] and the NRS [37, 38] are quite specific for measuring the severity of symptoms, and could be useful in combination with HRQL tools measuring issues like emotional or social well-being. The EORTC QLQ-C30 [19] is designed to cover issues relevant for cancer patients and may be a good generic measure to be completed by an item list consisting of the key DTF-specific issues identified in our focus groups, in order to create a more holistic perspective of HRQL issues in patients with DTF.

The results of the literature review show that researchers are interested in measuring the effect of DTF and its treatment on functioning or pain, but no consensus exists with respect to the preferred tool, as a DTF-specific tool has not been developed yet. One could argue that a combination of the aforementioned scores could be sufficient to get a clear view of relevant issues of DTF patients. A downside to this might be that patients are exposed to a large number of questions, which could be non-relevant and give patients an additional burden. A carefully developed DTF-specific tool could be effective in measuring HRQL.

There are limitations to the current systematic literature review. Since DTF is a rare soft tissue tumour, included studies comprise retrospective, small-sized studies with low methodologic quality. Additionally, risk of bias could not be assessed properly.

To create a HRQL tool which is suitable for DTF patients and to achieve at least satisfying content validity, focus groups were used which encouraged participants to discuss their views on HRQL issues [44]. Our focus group results suggest that patients with DTF often face problems with recognition and management because of the lack of diagnostic awareness, as a result of its rarity, and because of the striking discrepancy between its benign histological appearance and its local aggressive behaviour. This study identified key issues in six themes: (1) diagnosis, (2) treatment, (3) follow-up and recurrence, (4) physical domain, (5) psychological and emotional domain, and (6) social domain, which will be the basis of a future DTF-specific tool. The first three themes (diagnosis, treatment, and follow-up and recurrence) can be clustered as “the process of healthcare” and the last three themes (physical domain, psychological and emotional domain, and social domain) can be clustered as “symptoms and function”. We do acknowledge the overlap that can occur between themes.

The need to gain more insight into HRQL of DTF patients is reflected by several attempts made around the world. In the USA, the Desmoid Tumor Research Foundation (DTRF) patient registry opened recently (September 2017) to register clinical, pathological, and geographical variables of DTF patients. Additionally, a survey, based on both validated and non-validated HRQL questionnaires, was put together to gain more insight in HRQL of DTF patients [45]. The latter, a PRO-specific DTF tool, was presented on the Annual Meeting of the American Society of Clinical Oncology of 2017 [46]. In the Royal Marsden UK, two focus group sessions took place in March 2017 (Husson et al. 2018, manuscript submitted). This resulted in four key themes (diagnostic pathway, treatment pathway, living with DTF, supportive care). We found an interesting difference in the impact of DTF between the Dutch and UK focus group participants. Apart from the selection bias, which could be explained by the selection of patients and the willingness of patients to participate in such a study, and differences in the way patients had been treated with more often chemotherapy (Caelyx) in the UK focus group, other factors may play a role, which are beyond the individual patient level of these focus group participants. An international desmoid population-based questionnaire study could ideally give more detailed information. Such a study could also examine which patients are particularly at risk for poor disease-related outcomes on their quality of life.

Our focus group study has several limitations. First, the recruitment of participants for focus group sessions might have led to selection bias. Patients who are introvert, or who have minor symptoms, or received successful treatment might have been less likely to agree to participate in a focus group session and vice versa. A frequently heard response, when being approached for participation, was the worry about being influenced by negative experiences of other patients. However, in that case, most patients were willing to do a private face-to-face interview with the author to share their experiences. This suggests that not all patients feel comfortable to join a group session. The second limitation involves the small number of DTF patients. Due to the rarity of DTF, larger sample sizes are difficult to obtain in a single-centre study. Nevertheless, the small sample size gave all participants enough time to share their experiences [44]. The third limitation comprises the heterogeneity of the focus group participants, since we did not select participants based on their stage of disease or their treatment. Only one out of fifteen participants received previous systemic treatment, which might be an underestimation of the total percentage of patients in the DTF population receiving medication. We do acknowledge that every treatment modality (e.g. surgery, radiotherapy, chemotherapy) could impact HRQL on the short- and the long-term. However, regardless of previous treatments, patients, included in the focus groups, shared a wide variety of experiences coinciding with the chronic nature of the disease. This resulted in the report of various HRQL issues, which we believe do represent the entire spectrum of HRQL issues experienced by the DTF population.

To our knowledge, this is one of the few studies that explored currently used HRQL tools and the experience of HRQL issues in the setting of sporadic DTF. The strength of our study is the approach according to the EORTC guidelines for developing questionnaire modules [47]. By conducting the systematic literature review, we revealed the necessity for measuring HRQL outcomes in clinical practice and exposed a deficit in suitable HRQL tools for this patient group. The focus group approach elicits patients to explore and to clarify individual and shared perspectives. This resulted in the identification of key issues experienced by DTF patients and ensures the achievement of high content validity.

The results of the systematic literature review and the focus group sessions will be used to create a provisional list of issues which will be ranked by both patients and healthcare professionals for their relevance. Next, an item list will be created which will form the basis of the DTF-specific tool. This tool could complement the EORTC QLQ-C30 questionnaire with questions capturing issues raised from the focus groups, such as concerns about recurrences and emotional or psychological problems, and site-specific issues (i.e. extremity, abdominal wall). This questionnaire is much needed in order to understand effects of DTF and its treatment on patient-reported outcomes and provide support for patients who experience problems regarding physical, emotional, social, and psychological well-being. Also, knowledge about HRQL outcomes can be used for informed decision making during the diagnosis and treatment trajectory of this patient group.

## Conclusion

A DTF-specific tool and consensus regarding the preferred measurement tool for measuring HRQL in DTF patients is lacking in the literature. Used questionnaires either focus on single items, excluding possible items of significance, or are too generic. Existing questionnaires could be complemented with questions regarding key HRQL issues, identified during the focus group sessions, which DTF patients experience in various HRQL domains. This DTF-specific tool, validated in a large population study, would provide guidance for clinical practice, can compare treatment effects on HRQL and raise awareness of the impact of DTF on patients’ life.

## Appendix 1: Literature search 6 November 2017 (Embase.com)

(‘desmoid tumor’/exp OR Fibromatosis/exp OR ‘familial colon polyposis’/exp OR (desmoid* OR Fibromatos* OR ((familial* OR heredit* OR genetic* OR Adenomatous*) NEAR/6 polypos*)):ab,ti) AND (‘quality of life’/exp OR ‘quality of life assessment’/exp OR ‘functional assessment’/exp OR ‘general health status assessment’/exp OR ‘health status’/exp OR ‘health impact assessment’/de OR ‘daily life activity’/exp OR ‘ADL disability’/exp OR ‘patient satisfaction’/exp OR ‘distress syndrome’/exp OR ‘stress’/exp OR emotion/exp OR ‘sexuality’/exp OR ‘self concept’/exp OR ‘family relation’/exp OR ‘family life’/exp OR ‘coping behavior’/exp OR ‘disability’/de OR invalidity/de OR ‘immobility’/de OR ‘esthetics’/de OR ‘pain assessment’/exp OR ‘pain measurement’/de OR ‘social interaction’/exp OR ‘social life’/exp OR ‘social environment’/de OR ‘psychosocial environment’/de OR ‘social support’/de OR ‘social stress’/de OR ‘social rejection’/de OR ‘mental health’/exp OR ‘wellbeing’/exp OR ‘interview’/exp OR ‘questionnaire’/exp OR ‘assessment of humans’/exp OR ‘psychological aspect’/exp OR ‘psychology’/exp OR ‘marriage’/exp OR ((quality NEAR/3 life) OR hrql OR qol OR (Functional* NEAR/3 (outcome* OR asses*)) OR (daily NEAR/3 (life OR living)) OR ADL OR (patient NEAR/3 satisf*) OR ((health OR function*) NEAR/3 status*) OR eortc OR ((short-form OR sf) NEXT/1 (12 OR 20 OR 36)) OR sf12 OR sf20 OR sf36 OR distress OR (stress NEAR/3 (patient* OR personal* OR psycho* OR mental* OR life)) OR emotion* OR anxi* OR sexual* OR (self NEXT/1 (concept* OR esteem OR satisf* OR percept*)) OR body-image* OR burden* OR ((impact* OR problem* OR issue*) NEAR/6 (function* OR disease* OR personal* OR psycholog* OR body OR clinical* OR health* OR life OR daily OR tumor* OR tumour* OR social*)) OR psychosocial* OR worry* OR worrie* OR ((family OR interpersonal OR partner* OR spous*) NEAR/6 (relation* OR communicat* OR life OR involve*)) OR coping OR ((adaptive* OR adjustment*) NEAR/6 (behav* OR psycho*)) OR impairment* OR disabilit* OR invalidit* OR esthetic* OR aesthetic* OR cosmetic* OR beauty OR fitness OR (physical* NEAR/3 (condition* OR mobility)) OR immobility OR (pain* NEAR/6 (assess* OR inventor* OR measure*)) OR attractiveness* OR (social* NEAR/3 (isolat* OR distan* OR interact* OR life* OR support OR reject* OR participat* OR environment*)) OR feeling* OR (mental NEAR/3 (health OR status OR suffer*)) OR wellbeing OR well-being OR insecur* OR resilien* OR (symptom* NEAR/6 (assess* OR inventor* OR check*)) OR karnofsk* OR (karno* NEXT/3 (score* OR scale* OR perform* OR function* OR stat* OR index* OR rating)) OR (focus NEAR/3 group*) OR interview* OR questionnaire* OR (assessment* NEAR/3 human*) OR hopeless* OR fear OR frustrat* OR hopeless* OR helpless* OR unhapp* OR mood OR uncertaint* OR (lack NEAR/3 informat*) OR disturb* OR concerned OR deficit* OR ((self OR patient*) NEXT/1 report*) OR marriage*):ab,ti) NOT ([animals]/lim NOT [humans]/lim).
